# ‘The End of Sitting’: An Empirical Study on Working in an Office of the Future

**DOI:** 10.1007/s40279-015-0448-y

**Published:** 2015-12-17

**Authors:** Rob Withagen, Simone R. Caljouw

**Affiliations:** Center for Human Movement Sciences, University of Groningen, University Medical Center Groningen, PO Box 196, 9700 AD Groningen, The Netherlands

## Abstract

**Background:**

Inspired by recent findings that prolonged sitting has detrimental health effects, Rietveld Architecture Art Affordances (RAAAF) and visual artist Barbara Visser designed a working environment without chairs and desks. This environment, which they called The End of Sitting, is a sculpture whose surfaces afford working in several non-sitting postures (e.g. lying, standing, leaning).

**Objective:**

In the present study, it was tested how people use and experience The End of Sitting. Eighteen participants were to work in this environment and in a conventional office with chairs and desks, and the participants’ activities, postures, and locations in each working environment were monitored. In addition, participants’ experiences with working in the offices were measured with a questionnaire.

**Results:**

It was found that 83 % of participants worked in more than one non-sitting posture in The End of Sitting. All these participants also changed location in this working environment. On the other hand, in the conventional office all but one participant sat on a chair at a desk during the entire work session. On average, participants reported that The End of Sitting supported their well-being more than the conventional office. Participants also felt more energetic after working in The End of Sitting. No differences between the working environments were found in reported concentration levels and satisfaction with the created product.

**Conclusion:**

The End of Sitting is a potential alternative working environment that deserves to be examined in more detail.

## Key Points

**Table:** 

Recently, an office has been designed that lacks chairs and tables but consists instead of (slanted) surfaces that afford people to work in several non-sitting postures (e.g. standing, leaning, lying).
This newly designed office invites movement while working—83 % of participants worked in different non-sitting postures at different locations, giving rise to locomotion.
The ‘new’ office supported the well-being of participants more so than a conventional office, and had no negative effects on reported concentration levels and satisfaction with the produced work.

## Introduction

**Fig. 1:**
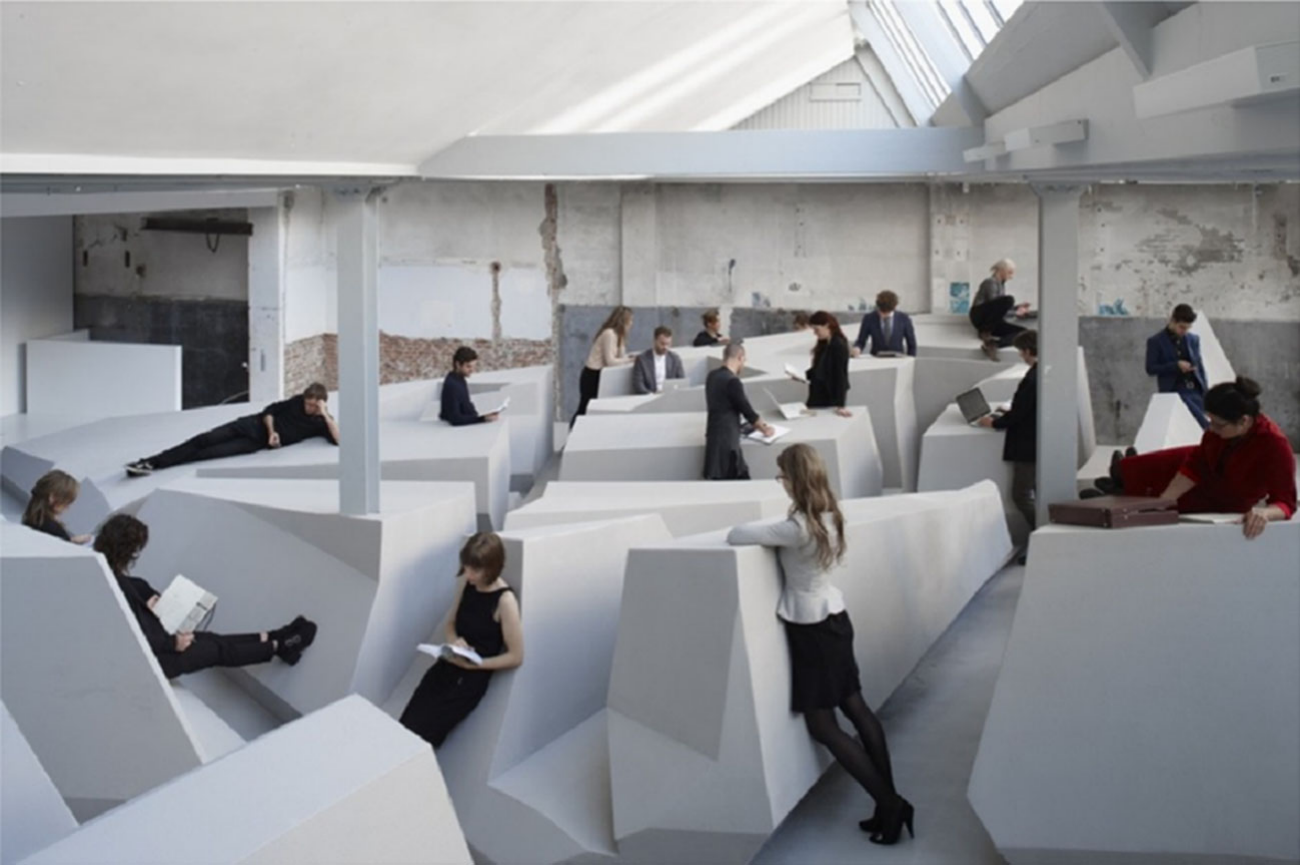
The end of sitting. The people in this photo did not participate in the study. Reproduced from Kempenaers [12], with permission

In the fall of 2014, Rietveld Architecture Art Affordances (RAAAF) and visual artist Barbara Visser realized a temporary office of the future in an exposition space in Amsterdam, The Netherlands. They were inspired by an article in the newspaper mentioning Hidde van der Ploeg’s scientific work on the negative health effects of sitting behavior. Van der Ploeg et al. [1] examined the relationship between sitting time and mortality and concluded that prolonged sitting is a risk factor for all-cause mortality. The evidence that sedentary behavior has detrimental health effects has recently been piling up [2–5]. For example, a recent meta-analysis demonstrated that even regular physical activity cannot annul the deleterious health consequences of prolonged sitting, although associations may become less pronounced as physical activity increases [6]. Accordingly, several activity-permissive furniture solutions for desk workers have been proposed to overcome these effects [7–9]. For example, active workstations have been realized in which people have a pair of pedals under their desk allowing them to ‘cycle’ while sitting on their chair. However, RAAAF and Visser opted for a more radical change of the working environment. They designed an office in which the chair and the desk are no longer the starting points [10, 11]. Instead, their office is a sculpture consisting of different surfaces that invite people to work in several non-sitting postures during the working day (see Fig. 1).

RAAAF and Visser, who called their installation The End of Sitting, were inspired by the concept of affordances. This concept was introduced in the 1960s by the ecological psychologist Gibson [13, 14] to refer to the action possibilities the environment offers us. For example, a chair affords sitting on, a cup affords grasping, and a ball affords catching or throwing. Since its inception, RAAAF used the concept of affordances as a starting point in their designs [15]. Indeed, if the environment consists of possibilities for action, then architectural interventions can be conceived as the creation of them. The End of Sitting offers a case in point. Indeed, RAAAF and Visser created an office consisting of several surfaces that afford people to work in standing, leaning, or lying^1^ postures (see Fig. 1). Because the designers intentionally created an environment that is comfortable but does not afford working comfortably in one posture for a long time, they expected people to move through the office and work in different postures during the day. Moreover, RAAAF and Visser created work surfaces of many different heights so that people can select a place in the working environment that fits their body size. After all, and as emphasized by Gibson [14], affordances exist by virtue of a relationship between the physical properties of the environment and the body [17–20]. RAAAF and Visser anticipated that it is the height of the supporting work surface relative to the height of the person that determines whether this surface affords working comfortably for him or her.

In the present study, we examined whether people used The End of Sitting as the designers intended. To this end, four specific questions were addressed: Which posture(s) do people work in? Do people work in the same posture or in different postures? Do they change location during the working session as the designers expected? Do people choose a work surface height that fits their body dimensions? In addition, we tested how people experience working in a non-sitting posture in this environment. To examine the potential benefits of The End of Sitting, the working behavior that is performed in this office will be compared with the behavior that takes place in a conventional office consisting of chairs and desks.

## Methods

### Participants

Eighteen participants (5 males, 13 females) between 19 and 28 years of age [mean age 21.7, standard deviation (SD) 3.0] volunteered to participate. The height of participants ranged from 164.5 to 204.0 cm (mean height 175.7 cm, SD 10.0 cm). All participants were enrolled in a university educational program or had completed one. All procedures followed were in accordance with the ethical standards of the Institutional Ethical Committee and the Helsinki Declaration of 1975, as revised in 2013. Informed consent was obtained from all participants included in the study.

### Design and Procedure

**Fig. 2:**
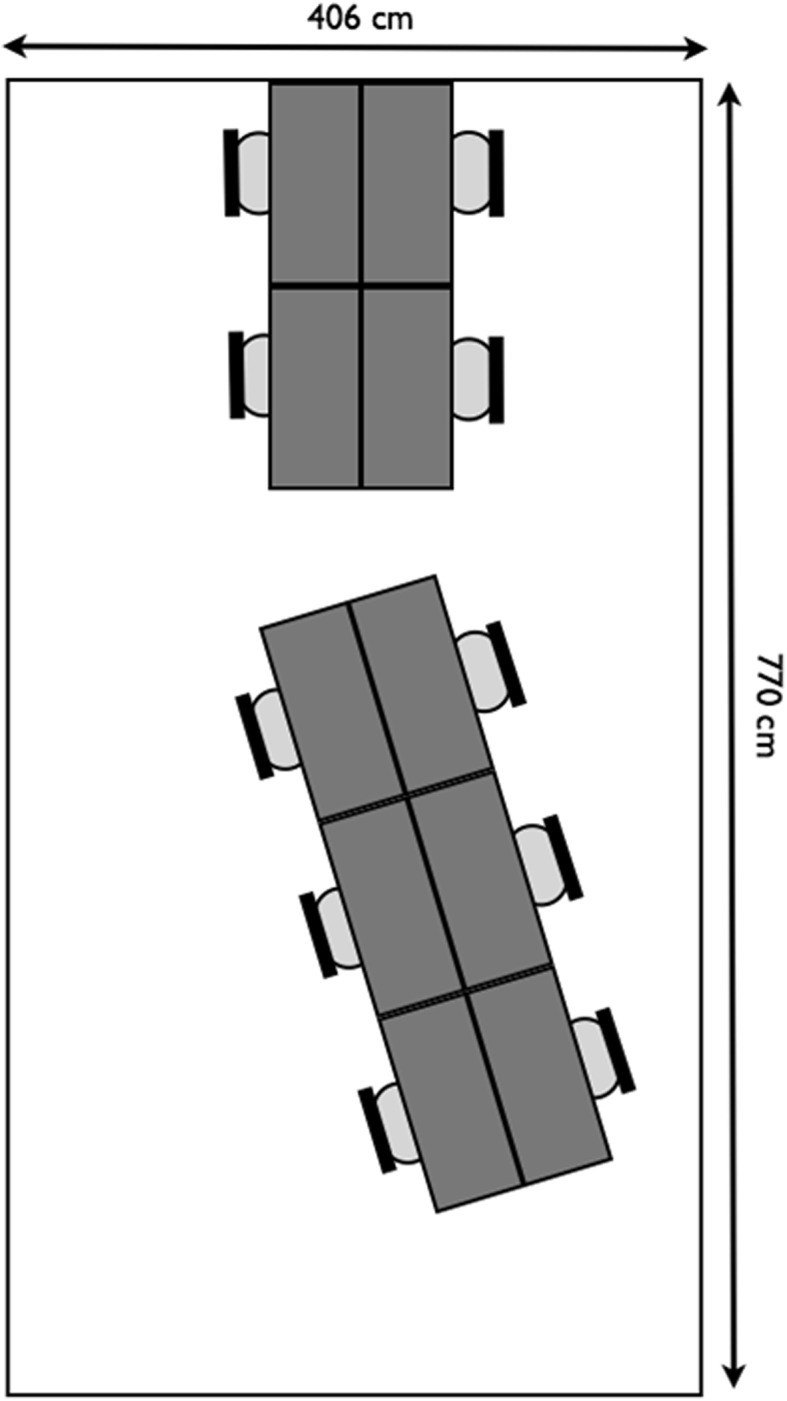
A top view of the conventional office. The surface of each desk was 107 × 46 cm

**Fig. 3:**
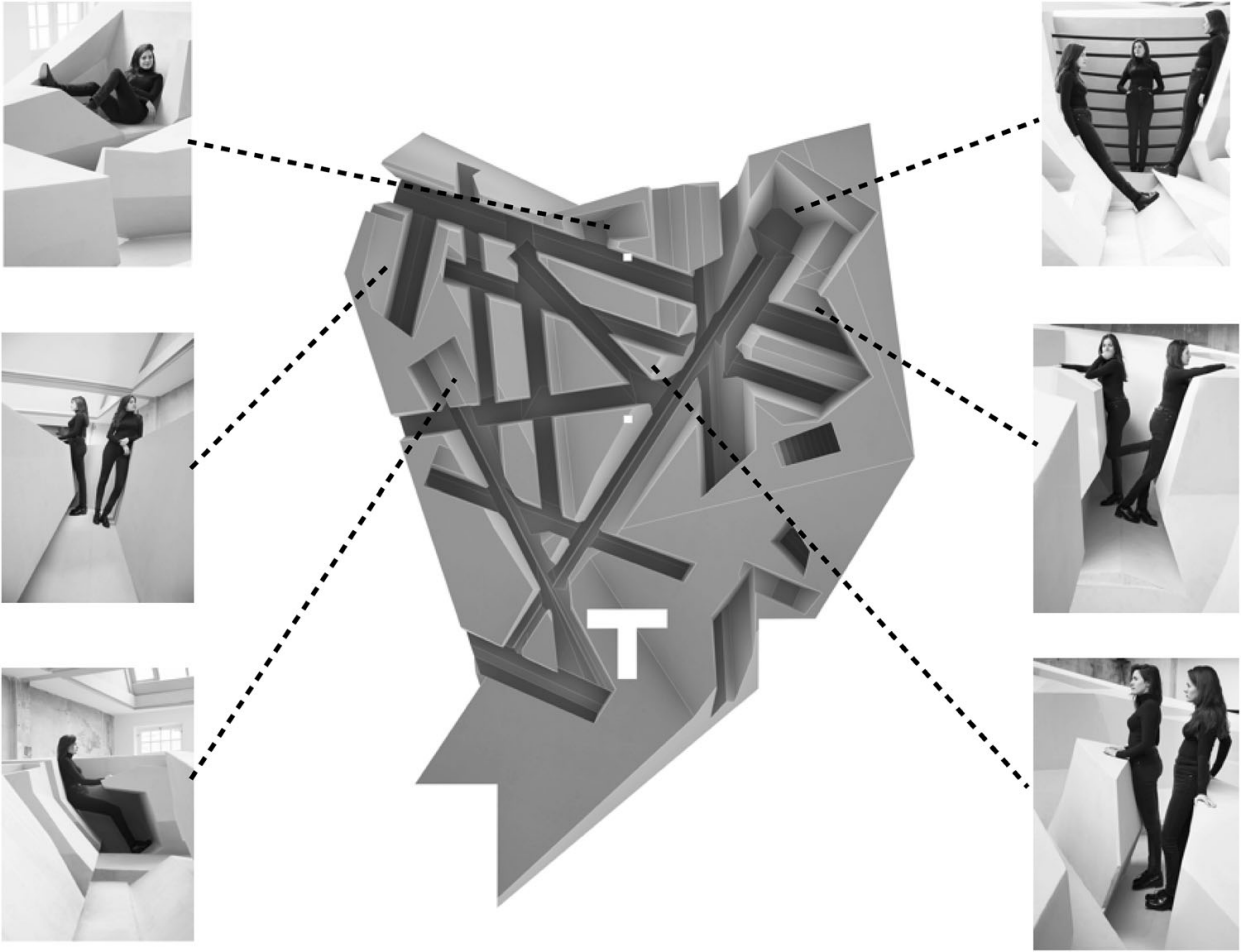
A top view of The End of Sitting, including the postures the surfaces afforded at different locations in the office. Reproduced from Rijkenberg [21], with permission. The office had a width of 13.5 m and a length of 21.9 m

Participants were to work in two different offices: The End of Sitting and a conventional office consisting of chairs and desks. Figures 2 and 3 depict top views of the offices in which the participants worked. The End of Sitting was realized in an exposition space in Amsterdam, with daylight coming from above and from one side of the room. On the other hand, the conventional office had large windows in one of the four walls of the room.

We created two groups of nine participants by randomly assigning participants to one of the groups. One group worked in the conventional office in the morning and in The End of Sitting in the afternoon, while the other group worked in the offices in the reverse order. Between the morning and afternoon working sessions there was a 2-h lunch break. In each office, participants were to make and prepare a 5-min oral presentation with slides of a chapter of a book on philosophy. In the morning session, both groups worked on the same 18-page chapter. In the afternoon session, another 18-page chapter of the book was used.

Participants were to finish preparing the presentation within 75 min. To ensure participants worked seriously, we told them before they started that one of them would be randomly selected to give the oral presentation to the other participants of the group at the end of the session. Before they started working they were free to explore both working environments for 10 min. After 40 min of working there was a mandatory 10-min break in which participants were provided with drinks and a little snack. There was then a 35-min working session, after which one participant was randomly selected to give the oral presentation to the other participants in his or her group. After this presentation, participants were asked to fill in a questionnaire on how they experienced working in the office. Because existing questionnaires on product design and comfort in offices typically include items on chairs and/or tables [22, 23], and thus are not suitable for measuring experiences while working in The End of Sitting, we created a questionnaire ourselves. This questionnaire consisted of 11 statements, and participants were asked to what extent these statements were applicable to working in the office using a 9-point Likert scale (see “Appendix”). Some of the included statements were selected from a validated questionnaire [23]. In the statements we added ourselves we aimed to do justice to the distinction that is made between comfort (well-being and aesthetics) and discomfort (biomechanics and fatigue) [23]. The items assessed feelings that people experience related to physical constraints (e.g. tired legs, posture), well-being (e.g. energetic, pleasantness) and aesthetics (the design is beautiful). Other items assessed estimated task performance (e.g. satisfaction with the prepared presentation, enough time available for preparing the presentation, able to collaborate and concentrate well). After participants completed the working sessions in both environments, we measured their body heights with a ruler.

Because we were interested in how people use and experience The End of Sitting for the activity it was designed for (i.e. working in non-sitting postures), we instructed participants not to sit on the top surface of the sculpture (this relatively flat surface allowed placement of a laptop or a book, and thus also afforded sitting). In the conventional office we put no restrictions whatsoever on the postures participants would like to work in. In both offices, participants were allowed to talk and work together on the presentation. To circumvent biases in the behavior that participants performed in the two offices, we told them that they were participating in a study on how productive people are in different offices.

### Analyses

The offices were equipped with video cameras that were used to record the sessions. The working sessions on these recordings were analyzed using the Observer XT Version 11.5 (Noldus Information Technology, Wageningen, The Netherlands). For each participant at each moment in time, we coded the location where they worked, the activity (categorized as reading the text, using the computer, talking, other, and not visible), and the posture in which they worked (categorized as sitting, leaning, standing, standing in a stooped position, lying on the back, lying on the belly, other, and not visible). Table 1 lists the operational definitions of the postures. To determine whether the behavioral criteria were sufficiently reliable, two observers (not the authors) independently coded the locations, postures, and activities of six randomly selected participants (33 % of the entire sample) in both working environments during the entire working sessions. The computed Cohen’s kappa demonstrated that the inter-rated reliability was good for location (0.990), activity (0.858), and posture (0.941).

**Table 1:** Operational definitions of the different postures that were coded

Categories	Operational definitions
Sit	Buttocks resting on horizontal surface with or without arm or upper body support
Lean	Buttocks resting on sloped surface and feet braced against floor or wall in front
Stand	Body in upright position with or without arm or body support
Stoop stand	Standing with inclined trunk with or without arm or body support
Lay back	Supine position with back support
Lay belly	Prone position with chest or arm support
Other	None of the above

## Results

### Activities

**Fig. 4:**
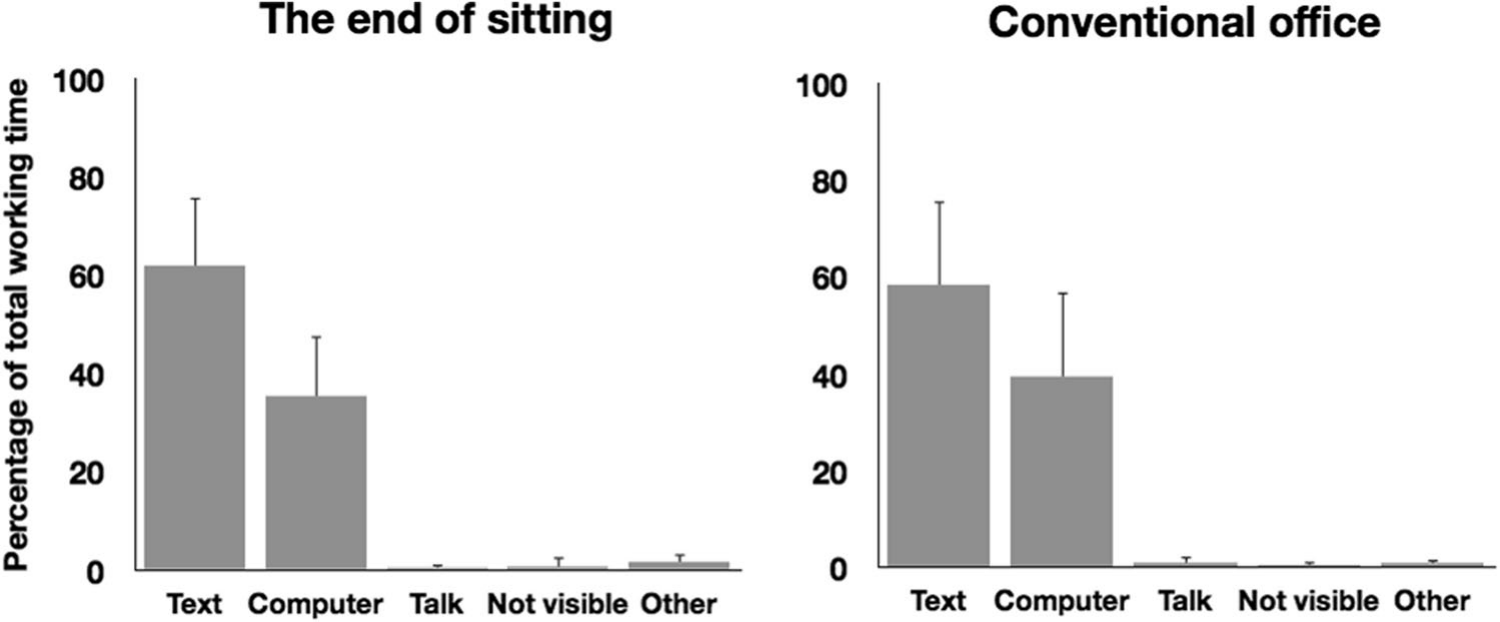
The time spent on different activities as a percentage of total working time. *Error bars* indicate one standard deviation. The *left-hand* figure depicts the activities in The End of Sitting, and the *right-hand* figure depicts the activities in the conventional office

For each participant, we computed the time they had spent on each activity (i.e. reading the text, using the computer, talking, other, not visible) as a percentage of the total working time in each session. For three participants, the activity that they were performing in The End of Sitting was not visible for more than 10 % of their working time;^2^ hence, we excluded these participants from this analysis. Figure 4 depicts the mean percentages of time spent on each activity in each office for the remaining 15 participants. Participants spent most of the time reading the text and using the computer; they hardly talked. We found no significant differences between the offices in terms of the percentage of time spent using the computer [*t* (14) = 1.78, *p* > 0.05], and reading the text [*t* (14) = 1.73, *p* > 0.05]. Apparently the time spent on the activities that are required to prepare an oral presentation of a book chapter was not different in the two offices.

### Postures and Locations

We computed the percentage of time each individual worked in the earlier enumerated postures (see Table 1) in each office. In the conventional office, the available chairs and desks were unsurprisingly used as objects to sit on and work at, respectively. All but one participant spent 100 % of the working time sitting on a chair. The participant who did not had spent 115 s reading while walking through the office, but worked in the same posture as the other participants for the remaining time.

**Fig. 5:**
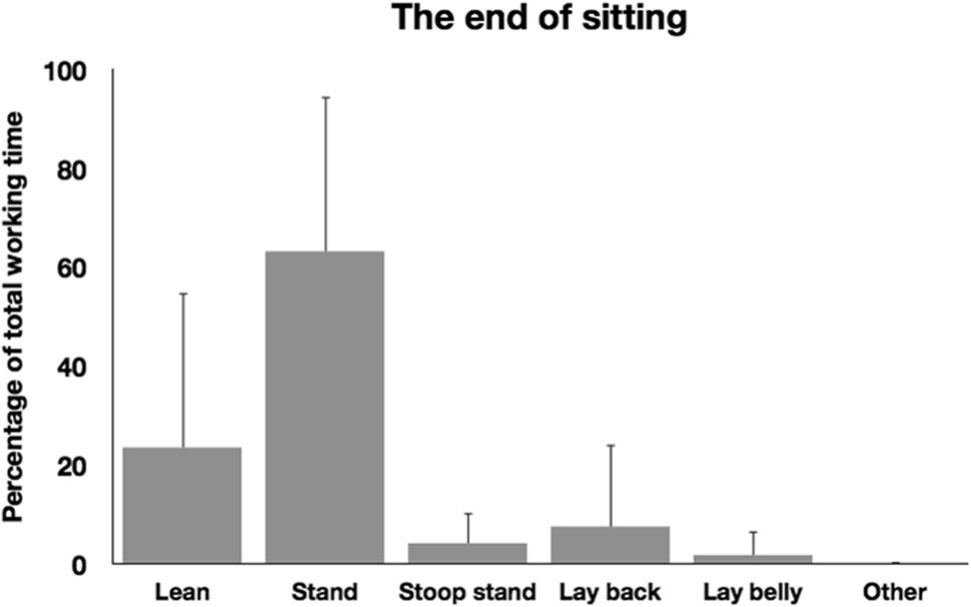
The time spent in different postures as a percentage of total working time in The End of Sitting. *Error bars* indicate one standard deviation

As mentioned in the introduction, The End of Sitting was designed to invite participants to work in different non-sitting postures during the working day. Participants indeed used several of the environment’s affordances while preparing the presentation (see Fig. 5). Although, on average, participants had spent some time in a lying and leaning posture, they worked most of the time standing. More interestingly, only 17 % of participants worked in just one posture while working on their presentations; 44 % worked in two postures, 17 % in three postures, and 22 % in four postures.

**Fig. 6:**
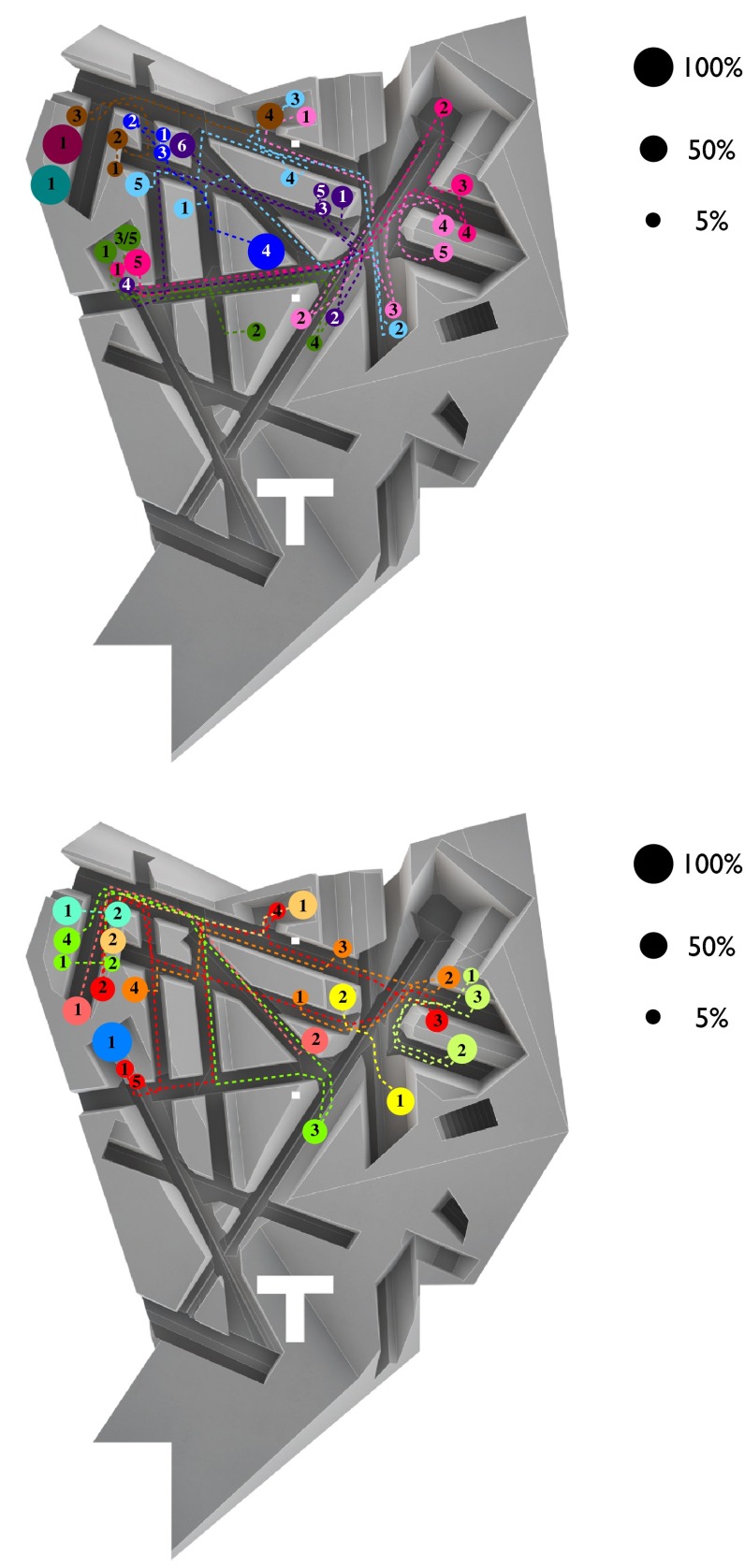
The location(s) at which each participant worked. Each *color* represents one participant. The *size of the circle* represents the time spent at the location, the *numbers in the circles* indicate the order in which the locations were taken by each participant, and the *dotted lines* represent the locomotion through the office. The *top* figure depicts the participants who worked in The End of Sitting in the morning, and the *bottom* figure depicts the participants who worked in the same office in the afternoon

All participants who worked in more than one posture (83 %) changed location during the working session, giving rise to locomotion through the environment (see Fig. 6). Thus, as RAAAF and Visser intended, in The End of Sitting the vast majority of participants indeed worked in different postures and changed location during the session.

### Preferred Height of Work Surface

RAAAF and Visser intentionally created work surfaces of different heights, allowing people to choose a surface that fits their body dimensions. To examine whether the chosen locations in the working environment were related to the participants’ heights, we determined, for each participant, the location at which he or she had spent most time working in a standing posture. Two participants did not work in a standing position, therefore they were not included in this analysis. A significant correlation was observed between the height of the chosen work surface and the height of the participant (*r* = 0.686, *p* < 0.01). The taller the person, the higher the work surface the person worked at in a standing position. On average, the height of the work surface was at 66 % of the body height (SD 5.8 %).

### Work Experience

As mentioned in the Methods section, we measured participants’ experiences working in the two offices with a questionnaire using a 9-point Likert scale . Table 2 lists the medians and the 25th and 75th percentiles of participants’ scores on each item of the questionnaire (see “Appendix”), in each office. Because several participants volunteered that they did not work together, and the above data analysis confirmed this, we decided not to include this item in our analysis. Wilcoxon signed-rank tests on participants’ scores on the Likert scale revealed no significant differences between the offices in terms of reported concentration levels, pleasantness of posture they worked in, and satisfaction with the created presentation (ps > 0.05). In addition, no differences were observed between the offices in terms of participant’s reports of whether they had sufficient time to prepare the presentation, and whether it was pleasant to have a break in the office (ps > 0.05). However, participants reported that they found it more pleasurable to work in The End of Sitting than in the conventional office (*z* = −2.56, *p* < 0.05), and that the former office supported their well-being more so than the latter (*z* = −2.77, *p* < 0.01). Interestingly, after working in non-sitting postures in the newly designed office, participants reported that they felt more energetic than after working in the conventional office (*z* = −3.45, *p* < 0.01), although their legs felt more tired (*z* = −3.54, *p* < 0.001). Participants also liked the design of The End of Sitting better than that of the conventional office (*z* = −3.53, *p* < 0.001). Apparently, compared with a conventional office with chairs and desks, working in one or more non-sitting postures in The End of Sitting had no negative effects on reported concentration levels and satisfaction with the prepared presentation, whereas it contributed to participants’ reported well-being and energy level.

**Table 2:** Medians (and 25th and 75th percentiles) of participants’ scores on the 9-point Likert scale for each item in each office

	Concentration	Pleasant*	Break	Nice posture	Well-being*	Energetic*	Design*	Tired legs*	Enough time	Satisfied presentation
End of sitting	7 (6.75–7)	7.5 (7–8)	7 (6–8)	7 (6–7)	7 (6–7)	7 (6–8)	7 (6–8.25)	4 (3–6.25)	4.5 (3.75–7)	6 (5–7)
Conventional office	7 (6–8)	6.5 (5–7)	6 (5–7)	6 (4–7)	6 (5–6.25)	4 (3–5)	3 (2–4.25)	2 (1–3)	4 (3–6.25)	6 (4.75–6.25)

In the items marked with an asterisk there was a significant difference between the two working environments

* *p* < .05

## Discussion

The present study examined how people use and experience The End of Sitting, a working environment that was designed by RAAAF and Visser. To examine the potential benefits of The End of Sitting, we let participants work in this office and in a conventional office, and monitored both the participants’ working behavior and their experiences in each office. Participants reported that The End of Sitting supported their well-being more so than the conventional office. In addition, participants reported that after working in the former office they felt more energetic than after working in the latter office. No differences between the offices were found in reported concentration levels and satisfaction with the prepared presentation. Interestingly, and as the designers intended, the vast majority of participants worked in different postures and changed location in The End of Sitting. On the other hand, in the conventional office all but one participant worked in a sitting posture.

These results suggest that The End of Sitting is an interesting alternative to the conventional office, and one that arguably promotes healthier behavior. As mentioned in the Introduction, several solutions to the detrimental health effects of prolonged sitting have been suggested; however, these solutions are often slight adjustments of the usual office furniture. Examples include a sit-to-stand adjustment to the desktop or a set of pedals fitted under the desk. However, preliminary indications suggest that simply placing a sit-to-stand desk may not be sufficient to invoke sustained clinically relevant decreases in the sitting time at work due to poor compliance in using them [24, 25]. Recent guidelines recommend that desk workers should avoid sitting for 2/8-h workday (progressing to 4/8-h workday), achieved by breaking up prolonged seating with bouts of low-intensity activity such as standing or slow walking [2]. Epidemiological data [26] and a number of recent intervention studies indicate that such interruptions of prolonged sitting improve biomarkers of health risk [27, 28], and reduce musculoskeletal discomfort [29]. A possible advantage of The End of Sitting to the earlier proposed activity-permissive solutions is that it does not afford working comfortably in one posture for a long time, thereby naturally inviting changes in postures and thus movement. Indeed, we found that even within the relatively short work session, many participants worked in several postures and changed location in the office.

Although the results of the present study seem promising, more research on The End of Sitting is needed to examine its overall effectiveness. Among other things, it is unclear whether people will still work in different postures when The End of Sitting becomes their permanent office. After all, in the present study, participants were to work in this office for only 75 min. Hence it might be that the changes in postures that we observed were due to a novelty effect, or that these changes reflect a person’s search for an optimal posture that she would work in for a long(er) time once it is found. Moreover, the people who participated in our study were relatively young, physically fit, and perhaps more open to new working environments than typical office workers. In addition, studies are needed to examine how productive people are while working in non-sitting postures in The End of Sitting. In the present study, no differences were observed between the two offices in the time spent reading and using the computer. In addition, no differences were found between participants’ reported concentration levels and satisfaction with their work. Taken together, these findings suggest that working in The End of Sitting does not have negative effects on productivity. However, the created product (a prepared oral presentation with slides) did not allow us to objectively determine productivity. Moreover, the two offices were created in different spaces, not allowing us to control several environmental factors (e.g. lighting conditions, acoustics) that might have an effect on the outcome measures of the questionnaire. For example, daylight conditions were different in the two offices (see the Methods section), and this factor has been found to have an effect on productivity as well as on feelings of well-being [30].

Longitudinal studies with typical office workers under controlled environmental conditions are needed to settle the above issues. Such studies are also required to examine the health effects of working in The End of Sitting. This working environment might not support the now heavily criticized prolonged sitting but this does not mean that the health effects of working in this environment are entirely positive. Perhaps working in the unusual postures that the office affords might lead to neuromuscular disorder or blood circulation problems in the long run.

## Conclusions

The present study revealed that The End of Sitting is used in the way its designers intended. The vast majority of participants worked in different postures and changed location during the working session. In addition, The End of Sitting supported the well-being of participants more so than a conventional office, and had no negative effects on reported concentration levels and satisfaction with the produced work. Although the overall effectiveness of The End of Sitting as a permanent office is not yet clear, our study suggests that such alternative working environments need to be taken seriously and deserve to be examined in full.

## Appendix

The translated questionnaire (original was in Dutch). Instruction to the participants: please indicate, by circling one of the nine digits, to what extent the following statements are applicable for working in the office space in which you have just made the presentation.

## Footnotes

1 Although lying is considered to be sedentary behavior [16], it is paradoxically afforded by The End of Sitting, an office that is designed to promote more healthy behavior.

2 There was one location in The End of Sitting that was not very well visible from the video recordings. Although at this location the posture of the participant could be observed based on the recordings, the activity could not. Hence, the three participants who worked more than 10 % of the working time at this spot were excluded from the analysis of the activity, but were included in the analyses of the locations and the postures.
